# Impact of Covid-19 on the Visit of Pediatric Patients with Injuries to the Emergency Department in Korea

**DOI:** 10.3390/children8070568

**Published:** 2021-07-02

**Authors:** Arum Choi, Woori Bae, Kyunghoon Kim, Sukil Kim

**Affiliations:** 1 Department of Preventive Medicine and Public Health, College of Medicine, The Catholic University of Korea, Seoul 06591, Korea; dyemelody@gmail.com; 2 Department of Pediatrics, College of Medicine, The Catholic University of Korea, Seoul 06591, Korea; baewool7777@hanmail.net

**Keywords:** COVID-19, pediatric emergency department, injury, emergency department utilization

## Abstract

The total number of pediatric emergency department (PED) visitors has decreased worldwide since the coronavirus disease (COVID-19) outbreak. We hypothesized that this might also affect the number of PED visits due to injuries. Therefore, we investigated these changes in PED visits after the COVID-19 outbreak through a long-term multicenter observational study. We assessed the changes in the proportion of injured pediatric patients’ weekly visits and the trend in the rate changes since the COVID-19 epidemic began by segmented regression analysis. We also evaluated the weekly change in the distribution of detailed diagnostic codes among pediatric patients with injuries before and after the COVID-19 pandemic. The proportion of injury-related PED visits increased when COVID-19 was first confirmed in Korea. After the COVID-19 epidemic, the proportion of foreign body ingestions and fracture patients among all pediatric patients with injuries increased significantly every week. The changes in the proportion of injured pediatric patients after the COVID-19 outbreak may have been the result of social distancing to prevent the spread of the virus. The risk of pediatric infections decreased but the risk of injury remained. Therefore, parents should take precautions to prevent infectious diseases and be careful to prevent children’s injuries at home.

## 1. Introduction

The severe acute respiratory syndrome caused by coronavirus 2 (SARS-CoV-2) originating from Wuhan, Hubei province, China in December 2019 [1] led the World Health Organization (WHO) to declare a coronavirus disease (COVID-19) pandemic on 11 March 2020 [2]. The first Korean case of COVID-19 was reported on 20 January 2020, and the number of patients has steadily increased. The third wave of the COVID-19 outbreak started in December 2020 and is still ongoing [3]. Vaccination programs began in March 2021, but the end of the COVID-19 pandemic is still unclear.

Regardless of the geographical distance from China, many people around the world including Korea, Japan, the United States, and European countries have rapidly become infected with the coronavirus [4]. To reduce the spread of COVID-19, many countries have implemented policies to restrict daily life such as social distancing and lockdowns [5,6,7]. South Korea has also implemented social distancing and wearing masks in public places such as buses, the metro, and workplaces. In addition, closing restaurants after 9 PM and restricting gatherings of more than five people were included in the precautionary measures [8].

As the prevalence of infectious diseases prevails, people become reluctant to engage in outside activities, especially attending hospitals, because of the potential infection risk. Previous studies reported that the average number of pediatric emergency department (PED) daily visits since the implementation of social distancing decreased by 63–75% compared to the same period in the previous year [9,10,11].

The decline in total patient visits to the PED due to social distancing after the COVID-19 outbreak is a global trend. We hypothesized that this may have affected visits to the PED of patients with injuries, based on previous publications [12,13,14]. Therefore, we investigated the changes in injury-related PED visits after the COVID-19 outbreak through a long-term multicenter observational study.

## 2. Materials and Methods

This was a retrospective observational study using electronic medical records (EMR) from six hospitals in large cities in South Korea, two tertiary hospitals in Seoul and Suwon, and four secondary hospitals in Seoul, Incheon, Bucheon, and Daejeon. The study subjects were patients under the age of 18 who visited PEDs from 1 January 2017 to 28 November 2020.

Those who had no diagnostic code, signs, and symptoms only (R code in the International Classification of Diseases (ICD)), visited for administrative purposes, or had missing values in the mode of arrival were excluded. Each single PED visit was considered an independent visit.

**Figure 1:**
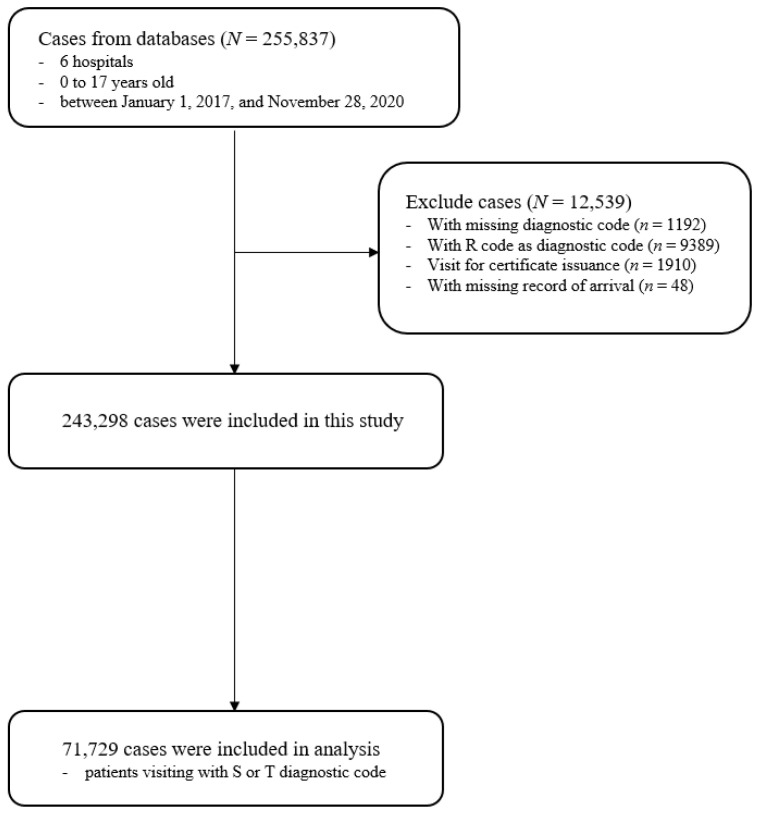
Flow chart of the study population.

During the study period, 255,837 patients visited the PEDs in six hospitals. After the application of the exclusion criteria, 243,298 cases were included. Among them, 71,729 patients had injuries coded to Chapter 19 of ICD-10 (Figure 1). 

The study variables included demographic variables, mode of arrival, disposition, and diagnoses coded according to the Korean modification of ICD-10 [15], which is called the Korean Standard Classification of Diseases-7 (KCD-7).

An injured patient was defined as a patient whose diagnosis was coded to Chapter 19 of ICD-10 (“Injuries, poisoning and certain other consequences of external causes, S00-T98”). Among all the injuries in Chapter 19, injury subgroups [16] were defined as in Table 1 and analyzed.

We investigated the trend in weekly pediatric injuries since the COVID-19 outbreak. We also assessed the change in the distribution of detailed diagnoses among injured pediatric patients before and after the COVID-19 outbreak.

We evaluated the differences in age, sex, the mode of arrival, and discharge between the years of comparison using a Chi-squared test. We performed segmented regression analysis [17] which is used to assess the effects of events in time series data. This shows the significance of the change in level and slope of the regression lines after the event. The event in this study is the outbreak of COVID-19 in Korea, the independent variable is the week of the year, and the dependent variable is the proportion of injured pediatric patients who visited the emergency departments. As seasonal variation existed in the visit trends, the data were adjusted for seasonality.

R version 4.0.0 (R Foundation for Statistical Computing, Vienna, Austria) was used for all statistical analyses.

## 3. Results

### 3.1. Demographic Characteristics of the Patients

After the COVID-19 outbreak in Korea, the number of total PED patient visits and injured patients decreased compared to the three previous years, the average number of monthly injury-related PED visits in 2020 decreased by 43.6% from 1677 to 945. Data for 2020 includes a month short of numbers, but a clear decline from the previous period was shown. However, there was an increase in the proportion of pediatric patients with injuries. In 2020, the proportion of patients with injuries increased by 8.6% from the average of the past three years. The proportion of males was 60.6% (Table 2).

### 3.2. Trends in Weekly Visits of Injured Patients 

**Figure 2:**
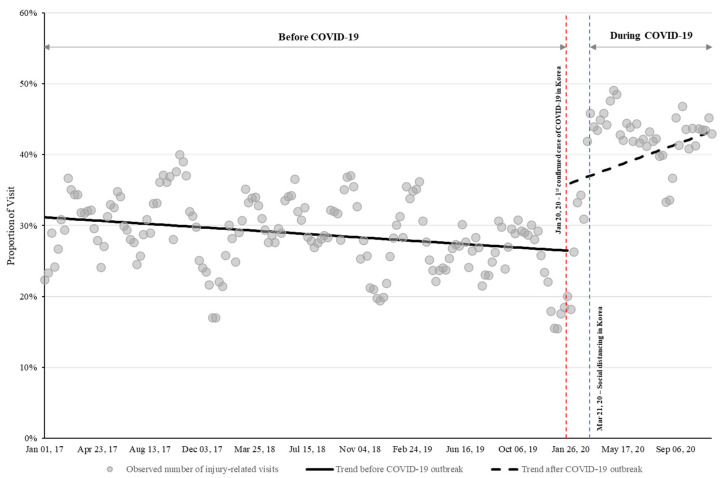
Segmented regression analysis of the weekly proportion of injury-related patient visits to PEDs. Compared to before the COVID-19 pandemic, the proportion of injury-related patient visits significantly increased (*p* < 0.001); COVID-19: coronavirus disease, PED: pediatric emergency department.

The influence of the COVID-19 outbreak on the number of injury-related PED visits was clearly revealed through segmented regression analysis. Before the COVID-19 outbreak, the proportion of injury-related visits decreased by 0.03% per week (*p* < 0.001, 95% CI: −0.04% to −0.02%) but increased significantly by 9.4% (*p* < 0.001, 95% CI: 6.9 % to 11.9%) at the outbreak, and then continued to increase by 0.2% per week (*p* < 0.001, 95% CI: 0.1% to 0.3%) (Figure 2, Table S1).

### 3.3. Sub-Group of Injured Patients and Trend in Foreign Body Ingestions, Fractures, and Burn Visits

The changes in the distribution of the detailed injury-related PED visits are shown in Table 3.

**Figure 3:**
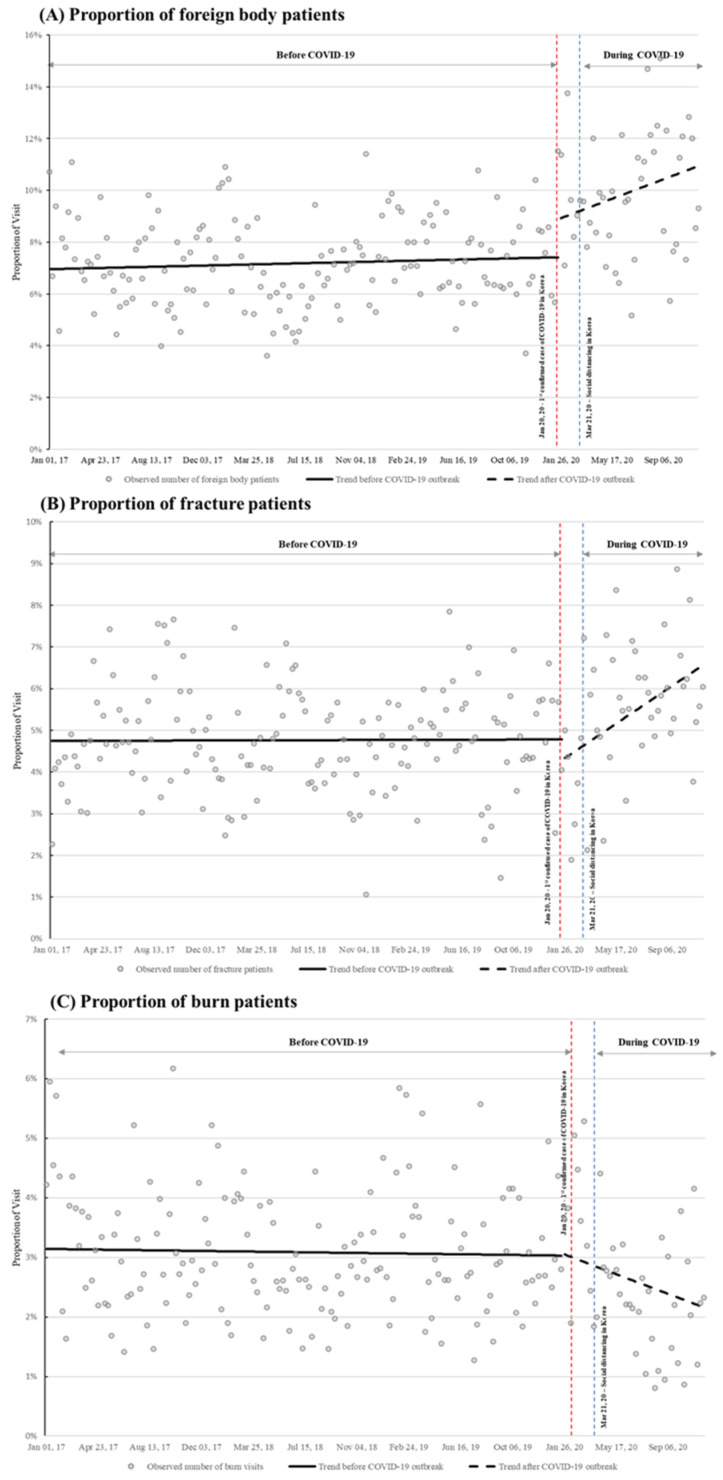
Segmented regression analysis of the weekly proportion of subgroups of injury-related visits. (**A**) Foreign body ingestion, (**B**) fractures, and (**C**) burns. Compared to before the COVID-19 pandemic, the proportion of foreign body ingestion and fracture patient visits significantly increased (*p* < 0.05, *p* < 0.001, respectively). The changes in the proportion of burn patients before and after COVID-19 was not statistically significant (*p* = 0.06); COVID-19: coronavirus disease, PED: pediatric emergency department.

After the COVID-19 outbreak, the proportion of foreign body ingestion and fracture patients among all pediatric patients with injuries significantly increased every week. The proportion of foreign body ingestions increased by 0.04% per week (*p* < 0.05, 95% CI: 0.01% to 0.1%) (Figure 3A, Table S2) and fractures increased by 0.1% per week (*p* < 0.001, 95% CI: 0.03% to 0.1%) (Figure 3B, Table S3). The number of burn injuries decreased by 3.3 persons per week (*p* < 0.05, 95% CI: −5.5 to 1.0), but the changes in the proportion of burn injury visits was not significant (Figure 3C, Table S4).

## 4. Discussion

The trend in injury-related PED visits analyzed in the current study changed after the COVID-19 outbreak. Since the first coronavirus case was confirmed, the number of visits to PEDs sharply decreased, and the number of injury-related PED visits also decreased. Subgroup analysis showed that the proportion of visits due to fractures and foreign body ingestions increased significantly.

COVID-19 changed peoples’ daily lives and many countries including Korea adopted social distancing policies to prevent the spread of COVID-19. As the prevalence of COVID-19 increased, people refrained from outside activities and stayed home. Especially, people became reluctant to visit hospitals due to the risk of infection. Our study also showed that the number of patients visiting PED in 2021 decreased by 43% compared to the last three years. Parents were not likely to visit PEDs if their children had minor health conditions or were in the early stages of an illness [18]. 

While decreases in PED visits in other countries were noticed after the introduction of social distancing [12,13,14], it began to drop in Korea just after the report of the first COVID-19 patient. Over 80% of Koreans started to wear masks and applied hand sanitizers at that time, as recommended by experts [19]. We think the first COVID-19 patient reminded Koreans of the MERS epidemic in 2015 [20] and they started to take precautions against transmission.

Children spent longer times at home because of the COVID-19 epidemic. Our results showed that, after the COVID-19 outbreak, the proportion of PED visits due to infectious diseases and respiratory diseases decreased, and the proportion of injury-related PED visits relatively increased. These findings may be the positive effect of the combination of hand-washing, wearing a mask, and school closures but 90% of children’s injuries occur in or around their home [21]. According to the locations of hazards by age group reported by the Korea Consumer Agency Consumer Injury Surveillance System (CISS), houses were ranked first as the places where injuries occurred in both children and teenagers. Moreover, in children younger than teenage years, houses accounted for 71% of the places where they were injured [22]. Before the COVID-19 outbreak, educational facilities and leisure & amusement facilities were the second most common places of injury following home. However, after the pandemic, the proportion of accidents in these places decreased due to social distancing and the proportion of accidents at homes increased [23]. In addition, the proportion of accidents on roads or sidewalks increased due to the increase in private car use and delivery services to avoid interpersonal contacts [24].

Since the COVID-19 outbreak, the burden of care in families has also increased as school closures and social distancing policies have meant that children are staying at home longer. This may also increase the risk of child abuse and other violence by increasing parental stress and family conflicts [25]. In the United States, the risk of family violence increased [26] and the proportion of ED visits related to child abuse and neglect per 100,000 cases increased [27] during the pandemic. In Korea, there has been no direct reports of increase in proportion of injury-related PED visits caused by child abuse after the COVID-19 outbreak. However, like other countries, the amount of child abuse and neglect might increase [25,27].

The proportion of pediatric patients with fractures and foreign body ingestions has increased. According to the Analysis of Child Safety Accident Trends in 2020 [23], the types of accident that occur mainly in children aged 0–14 in the past five years (2016 to 2020) are slipped, fell, or crashed accidents. However, there were an average of 2.5 cases per year related to hand sanitizer for children under the age of 14 from 2016 to 2019, but only 32 cases in 2020 when the COVID-19 disease spread, a 966.7% increase from the previous year. More than half of injuries associated with hand sanitizer were caused by eye entry or swallowing. The same trends were observed in other countries. Bram et al. [28] reported that there was a significant decrease in the incidence of pediatric fractures after the COVID-19 outbreak compared to pre-COVID-19 and the proportion of injuries occurring at home (32.5% to 57.8%) had increased, but those related to sports (26.0% to 7.2%) or playgrounds (9.0% to 5.2%) had decreased. In Italy, there was a dramatic increase in battery ingestions in children during the COVID-19 pandemic lockdown [29].

Some studies reported an increase in the number of burn patients. In the United Kingdom, after the spread of COVID-19, the incidence of burns in all ED visits [30] increased from 1.5% to 2.8%, and in the United States it increased from 0.48% to 0.94% of all ED visits [31]. These increases seem to be due to the secondary effect of having to go to a PED even for minor burns, because most primary care or local clinics were closed during the lockdown period. However, our results showed that there was a significant decrease in the number of pediatric burn patients after the COVID-19 outbreak but there was no significant change in the trend of burn injuries. Contrary to the UK and US, specific medical institutions were closed when COVID-19 patients were confirmed. But there was no lockdown of broad areas in Korea. This is considered to be the reason for the difference in burn incidences between Korea and other countries.

There were some limitations to this study. First, we collected the data from six hospitals so our results may not be generalizable to other hospitals in Korea. Nevertheless, the six hospitals are located in large cities where over 50% of the Korean population lives. And although the COVID-19 outbreak is progressing rapidly, it takes a long time to collect and analyze administrative data at the national level. Second, since the study used data provided anonymously, the possibility that information on the same patient was counted more than once could not be excluded. This is an inherent limitation of unidentified datasets. Third, if people continue to visit the PED for injury-related disease, but not to visit the PED for other diseases, the proportion of injury-related PED visits might increase. Since the denominator decreased, it is possible that the visits that were simply still seen in the PED were for injuries. This could be a marker of the fewer visits to the PED for other medical reasons than injuries. Lastly, this study was a retrospective observational study so we may not have collected all the variables we needed to account for the changes in injury-related PED visits. Therefore, further research is needed on where and why injuries occur specifically.

The strength of our study was that we used a large dataset from six hospitals over a period of four years including one year of data after the COVID-19 outbreak. The study results are reliable because they contain 240,000 cases controlling known seasonal variations in PED visits.

## 5. Conclusions

Our study results showed changes in the proportion of emergency department visits by pediatric patients with injuries after the COVID-19 outbreak. The number of visits to the PED declined noticeably in 2020, which seems to be the result of social distancing to prevent the spread of the epidemic. As the time spent at home instead of in outdoor activities increased, the risk of infection among children decreased, but the risk of injuries remained. Therefore, it is necessary not only to take precautions to prevent infectious diseases but also to be aware of possible injuries at home.

## Figures and Tables

**Table 1:** Subgroups of injury-related disease by ICD-10 code.

Diagnosis	ICD Code
Brain contusion	S06.1, S06.2, S06.3
Brain hemorrhage	S06.4, S06.5, S06.6, S06.7, S06.8, S06.9
Burn	T20-T32
Concussion	S06.0
Foreign body ingestions	T15-T19
Fracture	S02, S12, S22, S32, S42, S52, S62, S72, S82, S92, T02, T08, T10, T12, T14.2
Intoxication	T36-T65
Laceration	S01, S11, S21, S31, S41, S51, S61, S71, S81, S91, T01, T09.1, T11.1, T13.1, T14.1
Superficial injuries	S00, S10, S20, S30, S40, S50, S60, S70, S80, S90, T00, T09.0, T11.0, T13.0, T14.0

**Table 2:** Characteristics of Patients with injuries in the Pediatric Emergency Department, *N* (%).

Variables	2017	2018	2019	2020	*p* *
Total PED visits	70,888	73,234	68,469	30,707	<0.001
Injury-related visits	21,178 (29.9)	21,035 (28.7)	18,175 (26.5)	11,341 (36.9)	<0.001
Age					
0–11 months	1546 (7.3)	1505 (7.2)	1473 (8.1)	970 (8.6)	<0.001
1–2 years	6213 (29.3)	6023 (28.6)	4759 (26.2)	3090 (27.2)	
3–6 years	6322 (29.9)	6357 (30.2)	5380 (29.6)	3689 (32.5)	
7–17 years	7097 (33.5)	7150 (34.0)	6563 (36.1)	3592 (31.7)	
Sex					
Male	13,151 (62.1)	12,991 (61.8)	10,925 (60.1)	6873 (60.6)	<0.001
Mode of arrival					
Self-referred	20,326 (96.0)	20,108 (95.6)	17,240 (94.9)	10,744 (94.7)	<0.001
Referred from clinic	800 (3.8)	853 (4.1)	877 (4.8)	556 (4.9)	
Outpatient department	52 (0.2)	74 (0.4)	58 (0.3)	41 (0.4)	
Disposition					
Admission	566 (2.7)	547 (2.6)	558 (3.1)	332 (2.9)	<0.05

* *p*-values from the Chi-squared test.

**Table 3:** Changes in the detailed distribution of injury-related disease, *N* (%).

Diagnosis	2017	2018	2019	2020	*p* *
Brain contusion	22 (0.1)	64 (0.3)	19 (0.1)	5 (0.0)	<0.001
Brain hemorrhage	51 (0.2)	36 (0.2)	43 (0.2)	41 (0.4)	
Burn	681 (3.2)	593 (2.8)	572 (3.1)	294 (2.6)	
Concussion	2540 (12.0)	2538 (12.1)	2799 (15.4)	1679 (14.8)	
Foreign body ingestions	1495 (7.1)	1410 (6.7)	1368 (7.5)	1086 (9.6)	
Fracture	1055 (5.0)	969 (4.6)	882 (4.9)	624 (5.5)	
Intoxication	383 (1.8)	413 (2.0)	373 (2.1)	237 (2.1)	
Laceration	7211 (34.0)	7404 (35.2)	4938 (27.2)	3281 (28.9)	
Superficial injuries	4782 (22.6)	4640 (22.1)	4515 (24.8)	2713 (23.9)	
Others	2958 (14.0)	2968 (14.1)	2666 (14.7)	1381 (12.2)	

* *p*-values from the Chi-squared test.

## Data Availability

The data presented in this study are available on request from the corresponding author. The data are not publicly available due to ethical restriction.

## Supplementary Materials

The following are available online at https://www.mdpi.com/article/10.3390/children8070568/s1, Table S1: Segmented regression analysis of the weekly proportion of injury-related patient visits to PED. Table S2: Segmented regression analysis of the weekly proportion of foreign body ingestion patient visits to PED. Table S3: Segmented regression analysis of the weekly proportion of fracture patient visits to PED. Table S4: Segmented regression analysis of the weekly proportion of burn patient visits to PED.

## References

[1] Zhou P., Yang X.L., Wang X.G., Hu B., Zhang L., Zhang W., Si H.R., Zhu Y., Li B., Huang C.L. (2020). A pneumonia outbreak associated with a new coronavirus of probable bat origin. Nature.

[2] WHO WHO Director-General’s Opening Remarks at the Media Briefing on COVID-19—11 March 2020. https://www.who.int/director-general/speeches/detail/who-director-general-s-opening-remarks-at-the-media-briefing-on-covid-19---11-march-2020.

[3] CDC Coronavirus Diseases-19 Cases in Krea. http://ncov.mohw.go.kr/en/.

[4] Center C.R. https://coronavirus.jhu.edu/map.htm.

[5] Flaxman S., Mishra S., Gandy A., Unwin H.J.T., Mellan T.A., Coupland H., Whittaker C., Zhu H., Berah T., Eaton J.W. (2020). Estimating the effects of non-pharmaceutical interventions on COVID-19 in Europe. Nature.

[6] Mattioli A.V., Ballerini Puviani M., Nasi M., Farinetti A. (2020). COVID-19 pandemic: The effects of quarantine on cardiovascular risk. Eur. J. Clin. Nutr..

[7] Pulla P. (2020). Covid-19: India imposes lockdown for 21 days and cases rise. BMJ.

[8] KCDC COVID-19 Prevention Guidelines. http://ncov.mohw.go.kr/socdisBoardView.do?brdId=6&brdGubun=1.

[9] Li H., Yu G., Duan H., Fu J., Shu Q. (2020). Changes in children’s healthcare visits during coronavirus disease-2019 pandemic in Hangzhou, China. J. Pediatr..

[10] Dopfer C., Wetzke M., Zychlinsky Scharff A., Mueller F., Dressler F., Baumann U., Sasse M., Hansen G., Jablonka A., Happle C. (2020). COVID-19 related reduction in pediatric emergency healthcare utilization—A concerning trend. BMC Pediatr..

[11] Chaiyachati B.H., Agawu A., Zorc J.J., Balamuth F. (2020). Trends in pediatric emergency department utilization after institution of coronavirus disease-19 mandatory social distancing. J. Pediatr..

[12] Angoulvant F., Ouldali N., Yang D.D., Filser M., Gajdos V., Rybak A., Guedj R., Soussan-Banini V., Basmaci R., Lefevre-Utile A. (2021). Coronavirus disease 2019 pandemic: Impact caused by school closure and national lockdown on pediatric visits and admissions for viral and nonviral infections-A time series analysis. Clin. Infect. Dis..

[13] Ciofi Degli Atti M.L., Campana A., Muda A.O., Concato C., Rava L., Ricotta L., Reale A., Barbieri M., D’Argenio P., Lancella L. (2020). Facing SARS-CoV-2 pandemic at a COVID-19 regional children’s hospital in Italy. Pediatr. Infect. Dis. J..

[14] DeLaroche A.M., Rodean J., Aronson P.L., Fleegler E.W., Florin T.A., Goyal M., Hirsch A.W., Jain S., Kornblith A.E., Sills M.R. (2021). Pediatric emergency department visits at US children’s hospitals during the covid-19 pandemic. Pediatrics.

[15] Korea S. The 7th Korean Standard Disease Classification (KCD-7) Revision and Announcement. http://www.kostat.go.kr/portal/korea/kor_nw/1/15/index.board?bmode=read&aSeq=346904.

[16] Chen Y., Mo F., Yi Q.L., Jiang Y., Mao Y. (2013). Unintentional injury mortality and external causes in Canada from 2001 to 2007. Chronic. Dis. Inj. Can..

[17] Wagner A.K., Soumerai S.B., Zhang F., Ross-Degnan D. (2002). Segmented regression analysis of interrupted time series studies in medication use research. J. Clin. Pharm. Ther..

[18] Abela K.M., Wardell D., Rozmus C., LoBiondo-Wood G. (2020). Impact of pediatric critical illness and injury on families: An updated systematic review. J. Pediatr. Nurs..

[19] Association K.M. Advice for How to Use and Wear Masks. https://www.korea.kr/news/policyBriefingView.do?newsId=156375220.

[20] Oh M.D., Park W.B., Park S.W., Choe P.G., Bang J.H., Song K.H., Kim E.S., Kim H.B., Kim N.J. (2018). Middle East respiratory syndrome: What we learned from the 2015 outbreak in the Republic of Korea. Korean J. Intern. Med..

[21] Peden M., Oyegbite K., Ozanne-Smith J., Hyder A.A., Branche C., Rahman A., Rivara F., Bartolomeos K. (2008). World Report on Child Injury Prevention.

[22] CISS Consumer Risk Information Trend and Statistical Analysis in 2019. https://www.ciss.go.kr/www/selectBbsNttView.do?key=71&bbsNo=82&nttNo=34518&searchCtgry=&searchCnd=all&searchKrwd=&pageIndex=1&pageUnit=10&integrDeptCode=.

[23] Hazard Prevention Team (2021). Analysis of Child Safety Accident Trends in 2020.

[24] Kim C.M., Han A. (2020). Transport Policy after Covid-19 from Efficiency to Safety.

[25] Lee B., Jang H.S. (2021). Classifying latent types and identifying determinant factors for children’s experience of violence during the covid-19 pandemic. J. Korean Soc. Child Welf..

[26] Humphreys K.L., Myint M.T., Zeanah C.H. (2020). Increased risk for family violence during the covid-19 pandemic. Pediatrics.

[27] Swedo E., Idaikkadar N., Leemis R., Dias T., Radhakrishnan L., Stein Z., Chen M., Agathis N., Holland K. (2020). Trends in U.S. emergency department visits related to suspected or confirmed child abuse and neglect among children and adolescents aged <18 years before and during the covid-19 pandemic—United States, January 2019–September 2020. MMWR Morb. Mortal. Wkly Rep..

[28] Bram J.T., Johnson M.A., Magee L.C., Mehta N.N., Fazal F.Z., Baldwin K.D., Riley J., Shah A.S. (2020). Where have all the fractures gone? The epidemiology of pediatric fractures during the covid-19 pandemic. J. Pediatr. Orthop..

[29] Pizzol A., Rigazio C., Calvo P.L., Scottoni F., Pane A., Gennari F., Cisaro F. (2020). Foreign-body ingestions in children during covid-19 pandemic in a pediatric referral center. JPGN Rep..

[30] D’Asta F., Choong J., Thomas C., Adamson J., Wilson Y., Wilson D., Moiemen N., Farroha A. (2020). Paediatric burns epidemiology during COVID-19 pandemic and ‘stay home’ era. Burns.

[31] Sethuraman U., Stankovic C., Singer A., Vitale L., Krouse C.B., Cloutier D., Donoghue L., Klein J., Kannikeswaran N. (2021). Burn visits to a pediatric burn center during the COVID-19 pandemic and ‘Stay at home’ period. Burns.

