# Supplementary material for: Impact of Covid-19 on the Visit of Pediatric Patients with Injuries to the Emergency Department in Korea
Source: Children (Basel). 2021 Jul 2;8(7):568. doi: 10.3390/children8070568 (PMC8304024; doi:10.3390/children8070568)
Supplement: Supplementary file 1 [file children-08-00568-s001.zip › children-1250540-supplementary.pdf]

Table S1. Segmented regression analysis of the weekly proportion of injury-related patient visits to PED

Parameter	Coefficients	Standard errors	t-statistic	Confidence interval		p^*
Initial level	31.2%	0.6%	53.1	30.0%	32.4%	< 0.001
Before COVID-19 outbreak	-0.03%	0.01%	-4.7	-0.04%	-0.02%	< 0.001
Change in the level immediately after COVID-19 outbreak	9.4%	1.3%	7.4	6.9%	11.9%	< 0.001
During COVID-19 outbreak	0.2%	0.04%	4.5	0.1%	0.3%	< 0.001

* *p*-values from the segmented regression analysis.

Table S2. Segmented regression analysis of the weekly proportion of foreign body ingestion patient visits to PED

Parameter	Coefficients	Standard errors	t-statistic	Confidence interval		p^*
Initial level	7.0%	0.3%	27.5	6.5%	7.4%	< 0.001
Before COVID-19 outbreak	0.003%	0.003%	1.1	-0.002%	0.01%	0.3
Change in the level immediately after COVID-19 outbreak	1.5%	0.5%	2.7	0.4%	2.5%	< 0.05
During COVID-19 outbreak	0.04%	0.02%	2.3	0.01%	0.1%	< 0.05

* *p*-values from the segmented regression analysis.

Table S3. Segmented regression analysis of the weekly proportion of fracture patient visits to PED

Parameter	Coefficients	Standard errors	t-statistic	Confidence interval		p^*
Initial level	4.7%	0.2%	26.6	4.4%	5.1%	< 0.001
Before COVID-19 outbreak	0.0002%	0.002%	0.1	-0.004%	0.004%	0.9
Change in the level immediately after COVID-19 outbreak	-0.5%	0.4%	-1.3	-1.3%	0.3%	0.2
During COVID-19 outbreak	0.1%	0.01%	3.8	0.03%	0.1%	< 0.001

* *p*-values from the segmented regression analysis.

Table S4. Segmented regression analysis of the weekly proportion of burn patient visits to PED

Parameter	Coefficients	Standard errors	t-statistic	Confidence interval		p^*
Initial level	3.1%	0.1%	21.9	2.9%	3.4%	< 0.001
Before COVID-19 outbreak	-0.001%	0.002%	-0.5	-0.004%	0.003%	0.6
Change in the level immediately after COVID-19 outbreak	0.04%	0.3%	0.1	-0.6%	0.7%	0.9
During COVID-19 outbreak	-0.02%	0.01%	-1.8	0.04%	0.001%	0.1

* *p*-values from the segmented regression analysis.
